# Different Antigen-Specific CD4^+^ and CD8^+^ T-Cell Response against HCMV Proteins in Pregnant Women with Primary Infection and in Control Subjects with Remote Infection

**DOI:** 10.3390/jcm13185448

**Published:** 2024-09-13

**Authors:** Federica Zavaglio, Piera d’Angelo, Chiara Fornara, Paola Zelini, Giuditta Comolli, Milena Furione, Alessia Arossa, Arsenio Spinillo, Daniele Lilleri, Fausto Baldanti

**Affiliations:** 1 Microbiology and Virology Unit, Fondazione IRCCS Policlinico San Matteo, 27100 Pavia, Italy; f.zavaglio@smatteo.pv.it (F.Z.); p.dangelo@smatteo.pv.it (P.d.); p.zelini@smatteo.pv.it (P.Z.); g.comolli@smatteo.pv.it (G.C.); m.furione@smatteo.pv.it (M.F.); f.baldanti@smatteo.pv.it (F.B.); 2 General Clinical Laboratory, with Specialist Areas of Clinical Pathology, Microbiology and Virology, Istituti Clinici Scientifici Maugeri IRCCS, 27100 Pavia, Italy; chiara.fornara@icsmaugeri.it; 3 Obstetrics and Gynecology, Fondazione IRCCS Policlinico San Matteo, 27100 Pavia, Italy; a.arossa@smatteo.pv.it (A.A.); a.spinillo@smatteo.pv.it (A.S.); 4 Department of Clinical, Surgical, Diagnostic and Pediatric Sciences, University of Pavia, 27100 Pavia, Italy

**Keywords:** human cytomegalovirus, antigen-specific T-cell response, pregnancy

## Abstract

**Background/Objectives**: Human cytomegalovirus (HCMV) is the most frequent cause of congenital infections. The HCMV-specific T-cell response in primary infection may help define reliable correlates of immune protection in pregnancy. In this study, the antigen-specific T-cell response against different HCMV proteins (IE-1, pp65, gB, gHgLpUL128L) was investigated in pregnant women with primary infection and in control subjects with remote infection to identify possible components of a vaccine. **Methods**: Blood samples from 35 pregnant women with HCMV primary infection and 30 HCMV-seropositive healthy adult subjects with remote infection were tested. The antigen-specific T-cell response was measured using cytokine intracellular staining after stimulation with IE-1, pp65, gB and gHgLpUL128L peptides pool. **Results**: The pp65-specific CD4^+^ T-cell response was higher in pregnant women with HCMV primary infection at the late time point and in control subjects with remote infection, while the pregnant women at the early time point showed a higher gB-specific CD8^+^ T-cell response. Regarding the CD4^+^ and CD8^+^ T-cell phenotypes, we observed that HCMV-specific CD4^+^ and CD8^+^ T cells expressing CD45RA^+^ remained constant in pregnant women with primary infection at the early and late time points and in subjects with remote infection, while HCMV-specific CD4^+^ and CD8^+^ T cells expressing IL-7R^+^ or producing IL-2 were higher in control subjects with remote infection than in pregnant women with HCMV primary infection. **Conclusions**: The T-cell response was higher against gB in the early phase of infection and against pp65 in the late phase. Therefore, these proteins should be taken into consideration as candidates for a vaccine.

## 1. Introduction

Human cytomegalovirus (HCMV) is the most frequent cause of congenital infection, occurring in approximately 0.5–2% of pregnancies [1,2] and resulting in long-term sequelae in 20% of cases, including 13% of infants who are symptomatic at birth and 7% of infants who are asymptomatic at birth [3,4,5]. Among the most common long-term effects is sensorineural hearing loss (SNHL) [6,7,8,9,10,11]. According to observations, 7–21% of asymptomatic neonates with congenital infection have SNHL at 3–4 years old, and some children start showing symptoms at 7 years old or beyond [12].Congenital infection illness arising from primary maternal infection is frequently linked to more neurological damage and more severe SNHL, even if the incidence of SNHL in infants delivered to women with primary or non-primary infection is comparable [13,14,15,16]. The risk of congenital cytomegalovirus infection is around 40% after primary infection and much lower after non-primary infection [17,18,19,20]. The intrauterine transmission is associated with delayed development of the T and B cell response [21,22]; for example, the reduction in re-expression of CD45RA [23] on the surface of HCMV-specific effector memory T cells correlates with HCMV transmission to the fetus . Instead, a reduced risk of HCMV transmission to the fetus appears to be associated with the rapid development of HCMV-specific CD4^+^ T cells with a long-term memory phenotype, suggesting that IL-7R expression may be a predictive marker of protection [22]. In a previous study, we found that many years are required for the development of a long-term memory (LTM) response comparable to that of remote infection [24]. A HCMV vaccine should be preventing HCMV primary infection and repeated episodes of reactivation/reinfection and directly or indirectly protecting the two high-risk populations of pregnant women and immunocompromised patients [25]. In this way, the circulation of different HCMV strains may be reduced among pregnant women and immunocompromised patients [25].

In this study, we investigated the antigen-specific T-cell response against the non-structural protein IE-1 (produced in the immediate-early phase), the structural protein pp65 (internal tegument protein) and the envelope glycoprotein complexes, including the pentamer gHgLpUL128L and gB, in pregnant women at 2 and 12 months after HCMV primary infection and in control subjects with remote infection. We also investigated the correlation between the antigen-specific T-cell response and the protection against virus transmission to the fetus.

## 2. Methods

### 2.1. Subjects

Thirty-five pregnant women with HCMV primary infection were enrolled within three months after the onset of infection at Fondazione IRCCS Policlinico San Matteo, Pavia, Italy. Diagnosis of primary HCMV infection was based on two or more of the following parameters: presence of IgM, IgG seroconversion, low IgG avidity index, and detection of HCMV DNA in blood [26].

This retrospective study was performed according to guidelines and approved by the Ethics Committee and Fondazione IRCCS Policlinico San Matteo Institutional Review Board (Procedure number 20180075214). Participation in the study was discussed with the patient during the first clinical visit. All patients signed an informed consent. Data and clinical samples were coded to prevent the patient’s identity from being traced back to them and handled by doctors and investigators. Coded clinical samples were stored until the end of the study.

The participants were divided into two groups, those with HCMV primary infection and those with HCMV remote infection. Blood samples from 35 pregnant women with HCMV primary infection were collected and tested at the early time point (median time: 60; IQR 49–65 days after onset infection); 15 of them were also analyzed at a later time point (median time: 360; IQR 356–412 days after onset infection). In addition, 30 HCMV-seropositive healthy adult subjects (23 females and 7 males) with remote HCMV infection were enrolled as controls.

### 2.2. Diagnosis of HCMV

LIASON^®^ CMVIgMII (cat: 310755, Diasorin, Saluggia, Italy) assay was used to determine HCMV-specific IgM and values above 22 U/mL were considered positive; LIASON^®^IgGII (cat: 310745, Diasorin) assay was used to determine IgG antibody, the values above 14 U/mL were considered positive; LIASON^®^IgGAvII (cat: 310765, Diasorin) assay was used to determine IgG AI: AI < 0.15 was considered low, between 0.15 and 0.25 intermediate, and >0.25 high [26]. These kits detected antibodies specific for HCMV proteins pp150, pp52 and pp28.

Congenital HCMV infection was detected in two ways: either in the first trimester, 20–21 weeks of pregnancy, by HCMV DNA detection and virus isolation in amniotic fluid, and after delivery, by viral DNA detection in urine within two weeks after birth [26].

DNA was isolated from 200 µL of whole blood using QIAsymphony RGQ System and the QIAsymphony DNA Mini Kit (cat: 937236, QIAGEN, Hamburg, Germany). HCMV DNA amplification was performed with an in-house developed method targeting a region in the US8 gene [27]. Results were given as copies/mL (limit of detection 90 copies/mL).

### 2.3. Protein Peptides Pool

To evaluate the antigen-specific T-cell response, peptides pool representative of IE-1 (cat: P13202, JPT, Peptide Technologies, Berlin, Germany), pp65, gB, and gHgLpUL128L peptides pool (15 mers, overlapping by 10 amino acids; cat: na, all from A&A Labs LLC, San Diego, CA, USA), were used. A peptide pool of human actin (15 mers, overlapping by 10 amino acids; cat: na, Pepscan, Le-lystad, The Netherlands) was used as a negative control.

### 2.4. PBMC Isolation

Peripheral blood mononuclear cell (PBMC) were isolated from heparin blood, by standard density gradient centrifugation using Lymphoprep (cat: 1114547, Sentinel Diagnostics, Milano, Italy). Isolated PBMC were cryopreserved in RPMI-1640 (cat: ECB90062, Euroclone, Milano, Italy) supplemented with 10% dimethyl sulfoxide (DMSO) (cat: A3672,0100, PanReac AppliChem ITW reagents, Monza, Italy) and 25% human albumin (cat: na, Kedrion Biopharma, Lucca, Italy).

### 2.5. Stimulation with HCMV–Specific Peptides Pool and Cytokine Flow Cytometry Analysis

PBMC were stimulated for 16–18 h with peptide pools from IE-1, pp65, gB, gHgLpUL128L, and human actin [1 µg/mL] in the presence of 0.5 µg/mL of co-stimulator molecules, CD28 (clone CD28.2; cat: 555726, BD Biosciences, San Jose, CA, USA) and CD49d (clone 9F10; cat: 555502, BD Biosciences), and brefeldin A (cat: B7651-SGM, Sigma-Aldrich-Merck, Darmstadt, Germany) at a final concentration of 10 µg/mL. Cells were seeded in 96-well round bottom plates at a density of 0.5–1 × 10^6^ cells/200 µL culture medium per well. The culture medium was RPMI 1640 (Euroclone, Milan, Italy) supplemented with 100 U/mL penicillin, 100 μg/mL streptomycin (cat: ECB3001D, Euroclone), 2 mM L-glutamine (cat: ECB3000D, Euroclone), and 10% heat-inactivated FBS (cat: ECS1104L, Euroclone). PBMC were then incubated overnight at 37 °C with 5% CO_2_. MAb CD127 (IL-7R) PE (clone hIL-7R-M21; cat: 557938, BD Biosciences), which was added during the overnight incubation. Then, PBMC were washed with PBS (cat: ECB4004L, Euroclone) 2 mM EDTA (cat: 139-33-3, Sigma-Adrich-Merck) and stained with CD8 V500 (clone RPA-T8 ; cat: 560774, BD Biosciences) and CD197 (CCR7) BV421 (clone G043H7; cat: 353208, Biolegend, San Diego, CA, USA) in PBS 5% FBS for 30 min at 4 °C. Cells were then washed with PBS 5% FBS, fixed and permeabilized using Cytofix/Cytoperm (cat: 554722, BD Biosciences) for 20 min at 4 °C. Final staining with CD3 PerCP Cy 5.5 (clone UCHT1; cat: 560835), CD4 APC Cy7 (clone RPA-T4; cat: 557871), IFN-γ PECy7 (clone B27; cat: 557643), IL-2 APC (clone MQ1-17H12; cat: 554567) and CD45RA FITC (clone HI100; cat: 555488) (all from BD Biosciences) antibodies in Perm/Wash buffer 1× (cat: 554723, BD Biosciences) for 45 min at room temperature . Finally, cells were washed with Perm/Wash 1X and resuspended in PBS 1% paraformaldehyde (cat: 1.04002.1000, Sigma-Aldrich-Merck). Analysis was performed with FACS Canto II and FACS Lyric flow cytometer using FACSDiva™ v6.1.3 and BD FACSuite v.1.5 software (all from BD Biosciences). After the identification of memory T cells by exclusion of naïve T cells (CD45RA^+^ CCR7^+^), the percentage of IFN-γ producing CD4^+^ and CD8^+^ T cells was determined by subtracting the percentage of PBMCs incubated with human actin peptides from the percentage of PBMCs incubated with each HCMV proteins peptide pools, and among them the percentages of CD45RA^+^ or IL-7R^+^ or IL-2^+^ cells were calculated. Regarding IFN-γ CD4^+^ and CD8^+^ T cell response, a value < 0.05% antigen-specific T-cell was considered negative while a value ≥ 0.05% was considered positive.

### 2.6. Statistical Analysis

Statistical analyses were performed with GraphPad Prism 8.3.0 (GraphPad Software Inc., La Jolla, CA, USA). Comparison between three groups was performed using the Kruskal–Wallis test and Dunn’s post-test with correction for multiple comparisons, and two groups were compared using the Friedman test. The Mann–Whitney U-test was applied for unpaired comparison, while the Chi-square test was used to compare the difference in frequencies.

## 3. Results

### 3.1. HCMV-Specific T-Cell Response

**Figure 1:**
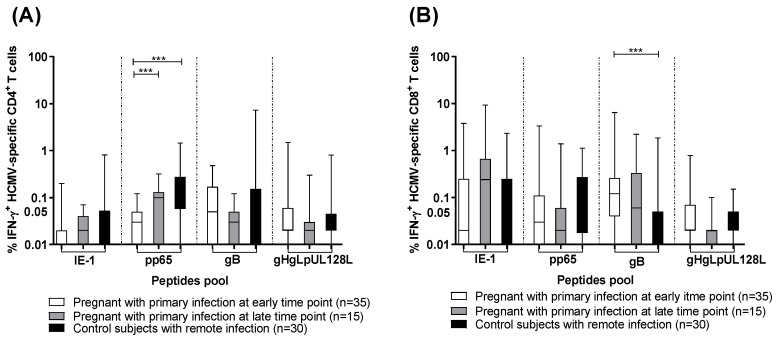
Percentage of HCMV-specific T cells producing IFN-γ after stimulation with IE-1, pp65, gB, gHgLpUL128L in pregnant women with HCMV primary infection at the early and the late time points, and in control subjects with HCMV remote infection. (**A**) Antigen-specific IFN-γ^+^ CD4^+^ T cells and (**B**) IFN-γ^+^ CD8^+^ T cells. Early time point: median: 60; (IQR49-65) days after onset infection. Late time point: median: 360; (IQR 356-412) days after onset infection. *** *p* < 0.001. Dash lines divide the different peptides pool.

The percentage of IFN-γ producing CD4^+^ and CD8^+^ T cells was calculated after stimulation with IE-1, pp65, gB, and gHgLpUL128L peptide pools in pregnant women with HCMV primary infection at an early and a late time point and in control subjects with HCMV remote infection. In particular, control subjects with remote infection and pregnant women with primary infection at the late time point developed higher levels of pp65-specific CD4^+^ T-cell response than pregnant women with primary infection at the early time point (*p* < 0.001) (Figure 1A). However, pregnant women with primary infection at the early time point showed higher gB-specific CD8^+^ T-cell response than control subjects with remote infection (*p* < 0.001) (Figure 1B). On the other hand, no difference was observed in the three groups for the HCMV-specific CD4^+^ and CD8^+^ T-cell responses detected by the other stimuli (Figure 1A,B).

### 3.2. Frequencies of Responders to Different HCMV Peptides Pool

**Figure 2:**
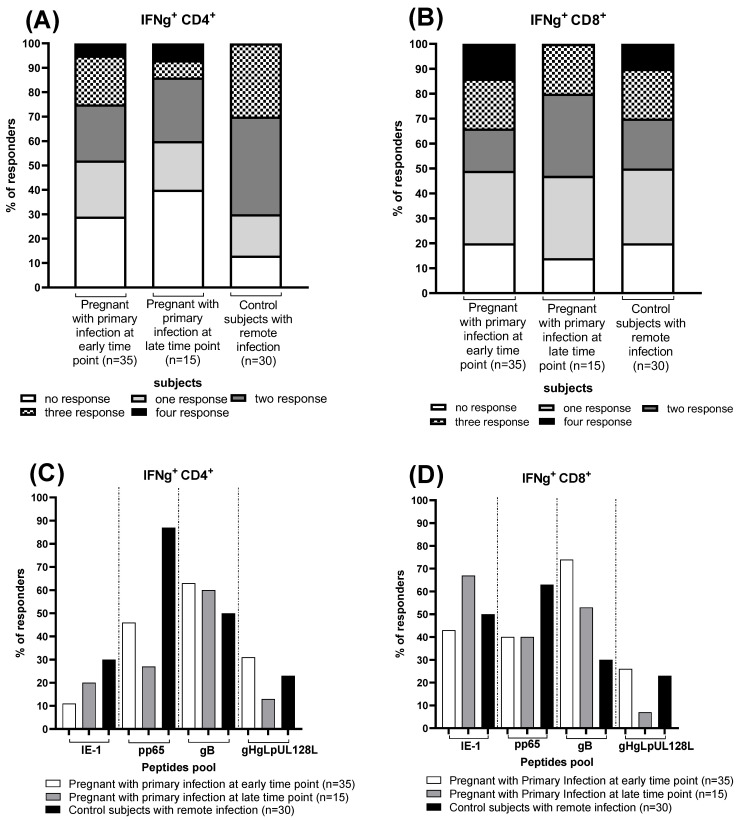
Frequency of CD4^+^ and CD8^+^ T-cell responders to HCMV peptide pools (IE-1, pp65, gB and gHgLpUL128L) detected in pregnant women with HCMV primary at the early and the late time point and in control subjects with HCMV remote infection. Percentage of responders to different numbers HCMV peptides pool (**A**,**B**) and frequencies of responders to IE-1, pp65, gB and gHgLpUL128L peptides pool (**C**,**D**), are reported. Early time point: median: 60; (IQR 49–65) days after onset infection. Late time point: median: 360; (IQR 356–412) days after onset infection. Dash lines divide the different peptides pool.

We calculate the number of pregnant women and controls responding to different stimuli, considering the IFN-γ CD4^+^ and CD8^+^ T-cell response negative with a *p* value < 0.05% and positive with a *p* value ≥ 0.05%. At the early time point, eight pregnant women (23%) showed a HCMV-specific CD4^+^ T-cell response against one or two peptide pools, seven (20%) showed a HCMV-specific CD4^+^ T-cell response against three peptides pool and two (5%) showed a HCMV-specific CD4^+^ T-cell response against four peptides pool, while ten (29%) showed no HCMV-specific CD4^+^ T-cell response (Figure 2A). Regarding the HCMV-specific CD8^+^ T-cell response, ten (29%) pregnant women showed a HCMV-specific CD8^+^ T-cell response against one peptide pool, 6 (17%) against two peptides pool, seven (20%) and 5 (14%) against three and four peptide pools, respectively, while seven (20%) showed no HCMV-specific CD8^+^ T-cell response (Figure 2B).

Among the fifteen pregnant women with HCMV primary infection at the late time point, we observed that three (20%) had the HCMV-specific CD4^+^ T-cell response against one peptide pool, four (26%) against two peptide pools and 1 (7%) against three and four peptide pools, while six (40%) showed no HCMV-specific CD4^+^ T-cell response (Figure 2A). For HCMV-specific CD8^+^ T-cell response, five (33%) pregnant women showed HCMV-specific CD8^+^ T-cell response against one and two peptide pools, 3 (20%) against the three peptides pool and nobody against four peptides pool, while 2 (14%) showed no HCMV-specific CD8^+^ T-cell response (Figure 2B).

Regarding the thirty control subjects with HCMV remote infection, six (17%) showed a HCMV-specific CD4^+^ T-cell response against one peptide pool, 12 (40%) and nine (30%) against two and three peptides pool, respectively, and nobody against four peptide pools, while three (13%) showed no HCMV-specific CD4^+^ T-cell response (Figure 2A). For HCMV-specific CD8^+^ T-cell response, nine (30%) control subjects showed a HCMV-specific CD8^+^ T-cell response against one peptide pool, 6 (20%) against two and three peptides pool and three (10%) against four peptides pool, while six (20%) showed no HCMV-specific CD8^+^ T-cell response (Figure 2B).

Regarding the different HCMV peptide pools, the percentage of responders for each group have been reported. About 70 percent of pregnant women at the early time point, have an HCMV-specific T-cell response for gB in both CD4^+^ and CD8^+^ T cells. The pp65 CD4^+^ and CD8^+^ T-cell response is present in about 40% of them, and the same percentage of women show a CD8^+^ T-cell response for IE-1. Most women with primary infection at the late time point show a CD8^+^ T-cell response for IE-1, present in about 70%, and for gB (~50%). The CD4^+^ T-cell response to gB is also present in a large part of them (~60%). Finally, analyzing the control group, it emerged that almost all of them (90%) develop a pp65 CD4^+^ T-cell response, while pp65 CD8^+^ T-cell response is found in nearly 70% of them. gHgLpUL128L-specific CD4^+^ and CD8^+^ T-cell response is the one found the less in all groups of subjects (Figure 2C,D).

### 3.3. CD45RA^+^ Effector Memory, IL-7R^+^ Long-Term Memory and IL2^+^ Producing CD4^+^ and CD8^+^ T Cells

**Figure 3:**
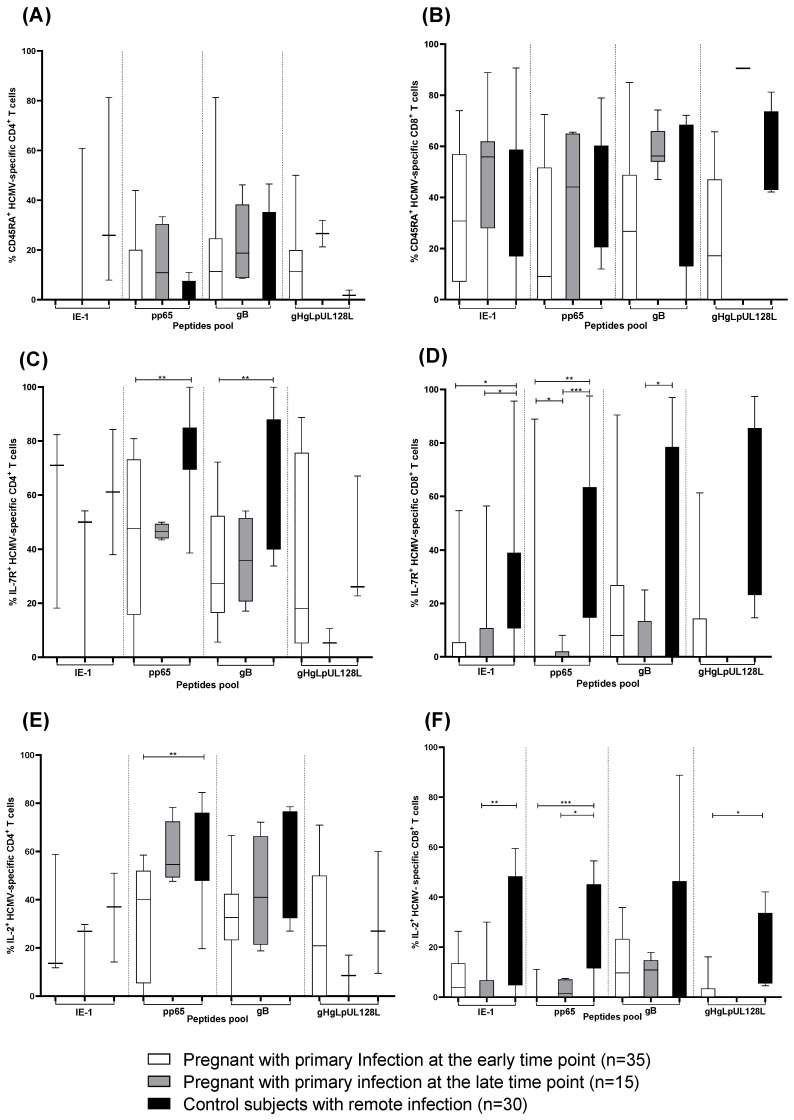
IE1, pp65, gB and gHgLpUL128L-specific CD4^+^ and CD8^+^ T cells expressing (**A**,**B**) CD45RA^+^, (**C**,**D**) IL-7R^+^ and (**E**,**F**) producing IL-2 in pregnant women with HCMV primary infection at the early and late time point and in subjects with remote infection. Early time point: median: 60; (IQR 49–65) days after onset infection. Late time point: median: 360; (IQR 356–412) days after onset infection. * *p* < 0.05, ** *p* < 0.01, *** *p* < 0.001. Dash lines divide the different peptides pool.

In addition to the HCMV-specific IFN-γ^+^ T-cell response, we analyzed the different CD4^+^ and CD8^+^ T-cell phenotypes. The percentage of IFN-γ producing CD4^+^ (Figure 3A) and CD8^+^ (Figure 3B) T cells showing a terminally differentiated effector memory phenotype (CD45RA^+^) remained constant in pregnant women with primary infection at the early and the late time points and in subjects with remote infection. The long-term memory phenotype of pp65 and gB-specific CD4^+^ T cells according to IL-7R expression was more represented in control subjects with remote infection than in pregnant women with primary infection at early and late time points (Figure 3C). Similarly, the percentage of IE-1, pp65, and gB-specific CD8^+^ T cells expressing IL-7R^+^ was higher in control subjects with remote infection than in pregnant women with HCMV primary infection at the early and late time points (Figure 3D).

The percentage of CD4^+^ T cells producing IL-2 was higher after stimulation with pp65 peptide pool in control subjects with remote infection than in pregnant women with HCMV primary infection at the early and late time points (Figure 3E). Instead, the percentage of CD8^+^ T cells producing IL-2 was higher after stimulation with IE-1, pp65, and gHgLpUL12L peptide pools in control subjects with remote infection than in pregnant women with HCMV primary infection at the early and late time points (Figure 3F).

### 3.4. HCMV-Specific T-Cell Response after Primary Infection and Virus Transmission to the Fetus

**Figure 4:**
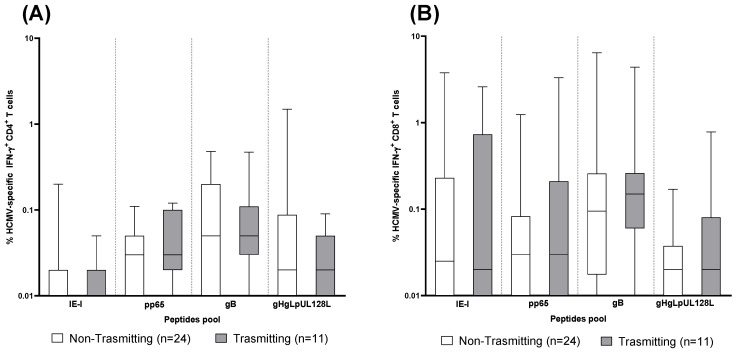
CD4^+^ and CD8^+^ T-cell response to human cytomegalovirus (HCMV) peptides pool proteins (IE-1, pp65, gB and gHgLpUL128L) detected in 24 non-transmitting and 11 transmitting pregnant women at the early time point. (**A**) IFNγ^+^ CD4^+^ T cells. (**B**) IFNγ^+^ CD8^+^ T cells. Early time point: median: 60; (IQR 49–65) days after onset infection.

To verify whether a particular antigen-specific CD4^+^ or CD8^+^ T-cell response is associated with a lower risk of virus transmission to the fetus, the percentage HCMV-specific IFN-γ^+^ CD4^+^and CD8^+^ T cells after stimulation with IE-1, pp65, gB and gHgLpUL128L peptide pools was compared between 24 non-transmitting and 11 transmitting pregnant women at the early time point (Figure 4). No difference was observed for any antigen-specific CD4^+^ or CD8^+^ T-cell response between non-transmitting and transmitting pregnant women (Figure 4A,B).

## 4. Discussion

In this study, we evaluated the antigen-specific T-cell response against different HCMV peptide pools (IE-1, pp65, gB, and gHgLpUL128L) to identify the ideal components of a vaccine. We analyzed pregnant women with HCMV primary infection at the early and late time points and control subjects with remote infection. We also investigated the correlation between the antigen-specific T-cell response and the virus transmission to the fetus.

In general, we observed that the antigen-specific CD4^+^ and CD8^+^ T-cell response was detected after IE-1, pp65, and gB stimulation. In particular, pp65 was the immunodominant target of CD4^+^ T cells in control subjects with remote infection, whereas gB was the immunodominant target of CD8^+^ T cells in pregnant women with primary infection at the early time point. Regarding the CD4^+^ and CD8^+^ T-cell phenotypes, we observed that HCMV-specific CD4^+^ and CD8^+^ T cells with a terminally differentiated effector memory phenotype (CD45RA^+^) remained constant in pregnant women with primary infection at the early and late time points and in subjects with remote infection, whereas HCMV-specific CD4^+^ and CD8^+^ T cells expressing IL-7R^+^ or producing IL-2 were higher in control subjects with remote infection than in pregnant women with HCMV primary infection. Regarding the risk of virus transmission to the fetus, no difference in HCMV-specific CD4^+^ and CD8^+^ T-cell response was observed between non-transmitting and transmitting pregnant women after stimulation with IE-1, pp65, gB, and gHgLpUL128L peptide pools.

In a previous study, the frequencies of antigen-specific T cell, in pregnant women with primary HCMV infection were determined using T cell libraries [22]. We showed that pp65 and gB proteins were recognized by CD4^+^ T cells, and IE-1 and pp65 proteins were recognized by CD8^+^ T cells; the pattern of T cell reactivity was the same in the early and late phases of infection [22]. Also, our results showed that pp65 was the immunodominant target for CD4^+^ in remote infection, whereas gB was the immunodominant target for CD8^+^ in primary infection. In another study, Fornara et al. used the cultured EliSpot to identify HCMV-specific T cells with proliferative capacity after stimulation with IE-1, IE-2, and pp65 peptides pools in pregnant women with primary HCMV infection [28]. Pregnant women tested in the second month after the onset of infection had a significantly lower response to the IE-1, IE-2, and pp65 than those with remote infection; however, pp65 was the immunodominant target of CD4^+^ T cells during primary infection [28]. The antigen-specific T-cell response after stimulation with IE-1 and pp65 peptide pools was also assessed in another study using the standard EliSpot assay [29]. The antigen-specific T-cell response to IE-1 and pp65 peptide pools was comparable at early and late time points was comparable [29]. It is also worth noting that the EliSpot assay targets both CD4^+^ and CD8^+^ T cells but does not discriminate between them. However, in some cases, it is important to distinguish between CD4^+^ and CD8^+^ T-cell responses. For example, in transplant patients, the long-term protection from HCMV infection is achieved when the CD4^+^ T-cell response is restored [30]; moreover, CD8^+^ T cells do not appear to be protective in the absence of the CD4^+^ T-cell counterpart [30].

The HCMV-specific CD4^+^ T cells expressing CD45RA^+^, measured by a flow-cytometry-based assay using HCMV-infected DC (CFC-iDC) as stimulus, showed a more rapid increase during the first month after primary infection [24], whereas the increase in CD8^+^ T cells expressing CD45RA^+^ was less evident [24]. In our results, the HCMV-specific CD4^+^ and CD8^+^T cells expressing CD45RA^+^, measured after stimulation with IE-1, pp65, gB, and gHgLpUL128L peptides pool, remained constant over time. This could be due to the small number of patients analyzed.

However, we observed that the HCMV-specific CD4^+^ and CD8^+^ T cells expressing IL-7R^+^ [31,32,33] or producing IL-2 after stimulation with IE-1, pp65, gB, and gHgLpUL128L peptide pools, showed an increase over time. The same results were observed in the other studies using HCMV-infected DC as stimulus [20,21,22]. Regarding the association between antigen-specific T cells and transmission of HCMV to the fetus, two previous studies showed that the frequencies of CD4^+^ and CD8^+^ T cells specific for IE-1, pp65, gB, and gHgLpUL128L were not different between transmitting and non-transmitting women [17].Similar results were found in our study. On the other hand, using cultured EliSpot, a significantly higher proliferative T-cell response was observed with pp65 but not with IE-1 or IE-2 [28].

The limitations of our study are the small number of patients examined, the wide range of days on which samples were taken from pregnant women with primary infection and collected in the early and late phases of infection, and the presence of both male and female control subjects with remote infection. The added value was the contemporary analysis of T-cell response against different HCMV peptide pool proteins and the CD4^+^ and CD8^+^ T-cell phenotypes.

Our study confirms no association between a particular antigenic specificity of the HCMV-specific T-cell response and transmission of HCMV to the fetus. The T-cell response was higher against gB and pp65 proteins in the early and late phases of infection in pregnant women with primary infection and in seropositive subjects, respectively, therefore, these proteins should be taken into consideration as candidates for a protective vaccine. The T-cell response is important in pregnancy and in immunocompromised patients, in particular the role of CD4^+^ T cells has become increasingly important. Immunocompromised patients who recovered both HCMV-specific CD4^+^ and CD8^+^ T cells were able to efficiently control HCMV replication in the blood [21,30,34,35,36,37,38]. In conclusion, a theoretically optimal recombinant HCMV vaccine composition should include the pp65 and gB proteins, which induce a protective T-cell response, and the pentameric complex gHgLpUL128L for the neutralizing antibody response [17,39,40].

## Data Availability

The data that support the findings of this study are available on request from the corresponding author. The data are not publicly available due to privacy or ethical restrictions.
